# Use of daratumumab in high risk multiple myeloma: A meta‐analysis

**DOI:** 10.1002/jha2.47

**Published:** 2020-07-02

**Authors:** Vikram Premkumar, Samuel Pan, Suzanne Lentzsch, Divaya Bhutani

**Affiliations:** ^1^ Columbia University Medical Center New York New York USA

**Keywords:** cytogenetics, immunotherapy, multiple myeloma

## Abstract

Daratumumab is approved for use in newly diagnosed and relapsed/refractory multiple myeloma (MM), however the patients most likely to benefit from its addition to standard anti‐myeloma therapy is unclear. This meta‐analysis included 2340 newly diagnosed MM patients (1982 with standard risk and 358 with high risk cytogenetics) and 673 patients with relapsed/refractory MM (513 with standard risk and 160 with high risk cytogenetics) to assess which cytogenetic subgroups derived PFS benefit from Daratumumab. Studies included were the CASSIOPEIA, MAIA and ALCYONE (for newly diagnosed MM) and the CASTOR and POLLUX trials (for relapsed/refractory MM). Daratumumab's addition led to a clear benefit in standard risk newly diagnosed MM (HR 0.43; 95% CI, 0.35‐0.53; *P* < .05) and both high and standard risk relapsed/refractory disease (HR 0.28; 95% CI, 0.21‐0.36; *P* < .05 and HR 0.48; 95% CI, 0.30‐0.76; *P* < .05, respectively). No clear benefit was seen in newly diagnosed high risk MM. These findings fail to demonstrate PFS benefit from Daratumumab's addition in high risk newly diagnosed MM. Data forthcoming from the GRIFFIN and MASTER trials may increase the power of the study and provide a definitive answer. Daratumumab remains important in standard risk upfront and relapsed/refractory MM and high risk relapsed/refractory MM.

## 1 INTRODUCTION

Among patients with newly diagnosed multiple myeloma (NDMM), nearly 20% present with high risk disease (HRD) features defined by cytogenetic/FISH analysis, namely del(17p) or p53, t(4:14) and t(14:16), with approximately 10% of patients meeting R‐ISS Stage III criteria [1]. HRD increases in incidence during subsequent relapses and despite the advent of novel therapies, patients with HRD have only attained a modest increase in overall survival (OS) [2].

Recently Daratumumab (Dara) has been approved in relapsed/refractory multiple myeloma (RRMM) as monotherapy and in combination therapy [3, 4, 5, 6, 7]. Similarly, Dara has gained approval in NDMM in combination therapy [8, 9, 10]. Most approvals are based on phase 3 randomized control trials (RCTs) meeting primary endpoints of progression‐free survival (PFS) improvement. The substantial financial implications and increased rates of adverse events like infections [3–5, 8–10] associated with Dara make it important to identify which patients, and in which treatment line, would derive the greatest benefit and whether Dara can overcome the poor prognosis of high‐risk patients.

Therefore, we conducted a meta‐analysis to evaluate the effect of Dara on NDMM and RRMM patients based on cytogenetic risk.

## 2 PATIENTS AND METHODS

### 2.1 Study identification and selection

Prior to performing a PubMed search, criteria for inclusion were defined as any RCT in NDMM or RRMM that included PFS data regarding well‐established standard of care myeloma therapy with and without Dara. In order to control for heterogeneity between studies only RCTs with both control and experimental arms (standard therapy vs Dara + standard therapy) would be considered. Given the primary endpoint of this meta‐analysis, studies were excluded if there was no stratification of PFS data by cytogenetic risk. A PubMed search using the keywords of “daratumumab,” “myeloma,” “newly‐diagnosed,” “relapsed,” and “refractory” revealed 10 studies with five meeting inclusion/exclusion criteria: CASTOR [4, 7], POLLUX [5, 6], ALCYONE [8], MAIA [9], and CASSIOPEIA [10] (Table 1). The other five studies did not have a control arm and/or did not stratify PFS by cytogenetic risk.

**TABLE 1:** Trial details, patient characteristics, and hazard ratios for PFS with 95% confidence intervals for patients with standard and high risk patients treated with or without daratumumab in the trials selected for this meta‐analysis

Trial Name	NCT number	Investigational Arm	Control Arm	Median Age in Years (range)	Median Prior Regimens, range	Primary Endpoint	HR for PFS in SRD	HR for PFS in HRD
NDMM								
ALCYONE^8^	NCT02195479	Dara + VMP	VMP	71.0 (40‐93)	0 (NDMM)	PFS	0.39 (0.28‐0.55), *P* < .05, n = 518	0.78 (0.43‐1.43), n = 98
CASSIOPEIA^9^	NCT02541383	Dara + VTD	VTD	58.0 (22‐65)	0 (NDMM)	sCR rate 100 days after ASCT	0.41 (0.26‐0.62), *P* < .05, n = 914	0.67 (0.35‐1.35), n = 168
MAIA^10^	NCT02252172	Dara + RD	RD	73 (45‐90)	0 (NDMM)	PFS	0.49 ( 0.36‐0.67), *P* < .05, n = 550	0.85 (0.44‐1.65), n = 92
RRMM								
CASTOR^4, 7^	NCT02136134	Dara + VD	VD	64 (30‐88)	2, 1‐10	PFS	0.26 (0.18‐0.37), *P* < .05, n = 258	0.45 (0.25‐0.80), *P* < .05, n = 95
POLLUX^5, 6^	NCT02076009	Dara + RD	RD	65 (34‐89)	1, 1‐11	PFS	0.30 (0.20‐0.47), *P* < .0001, n = 246	0.53 (0.25‐1.13), *P* = .0921, n = 65

Abbreviations: NDMM, newly diagnosed multiple myeloma; RRMM, relapsed/refractory multiple myeloma; NCT, National Clinical Trial; Dara, Daratumumab; V, bortezomib; M, melphalan; D, dexamethasone; P, prednisone; T, thalidomide; R, lenalidomide; PFS, progression‐free survival; HRD, high risk disease; SRD, standard risk disease; HR, hazard ratio.

The CASSIOPEIA trial was sponsored by The Intergroupe Francophone du Myélome (IFM) and Dutch‐Belgian Cooperative Trial Group for Hematology Oncology; all patients provided written informed consent. The MAIA, ALCYONE, CASTOR and POLLUX trials were sponsored by Janssen Research and Development and independent ethics committees or institutional review boards at each site approved the protocol. All patients provided written informed consent. All data were extracted solely from published reports (see references); no data were separately taken or requested from sponsors of the examined studies.

The CASSIOPEIA trial defined high risk MM as having at least one HRD cytogenetic abnormality: del(17p) in at least 50% of neoplastic cells or t(4;14) in at least 30% of cells. The MAIA, ALCYONE and CASTOR trials defined HRD as having at least one of the following abnormalities: del (17p), t(14;16), or t(4;14). The POLLUX trial defined HRD as having at least one of the following abnormalities: del (17p) in at least 50% of neoplastic cells or the presence of t(4;14) or t(14;16).

### 2.2 Outcome measures

The primary endpoint was PFS. Other analyses included high quality responses (≥VGPR) and minimal residual disease (MRD) negativity rate. For analysis, patients were divided into ND and RR.

### 2.3 Statistical analysis

We used R version 3.6.1 (R Foundation) and the “Meta” package (version 4.9–7). ND and RR studies were stratified and analyzed separately, and pooled odds ratios (ORs) using inverse variance method, and PFS hazard ratios (HRs), 95% CIs were calculated by DerSimonian and Laird random effects model. Statistical significance of pooled trials was assessed through weighted *Z*‐scores. Study biases were analyzed through funnel plots, a test for heterogeneity using Cochran's *Q* to verify if any studies were overly influential and the *i* ^2^ statistic to measure heterogeneity as low, moderate, or high.

## 3 RESULTS

We examined results for ≥VGPR, MRD negativity, PFS, and OS. Rates of ≥VGPR, MRD negativity and OS stratified by cytogenetic risk were not available in all studies and thus limited analysis to PFS.

**FIGURE 1:**
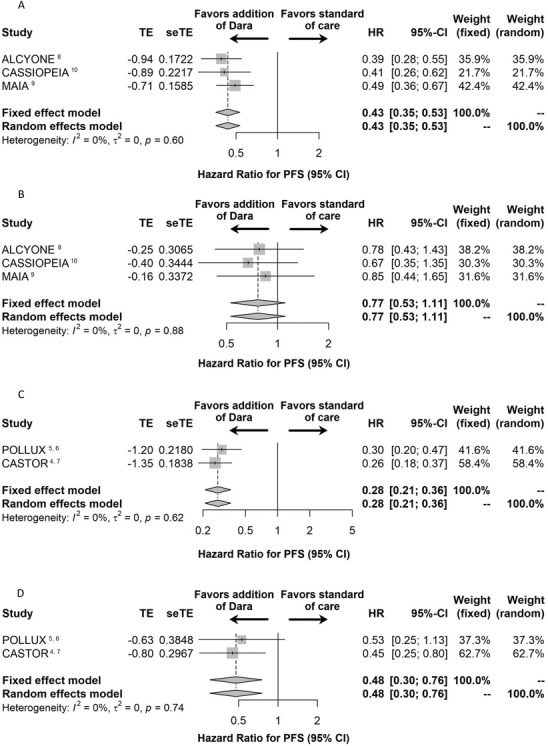
A–D, Forest plots for patients with NDMM and RRMM. A, NDMM patients with standard risk disease; B, NDMM patients with high risk disease; C, RRMM patients with standard risk disease; D, RRMM patients with high risk disease. Treatment effect (TE), treatment effect standard error (seTE), hazard ratios (HR), and their 95% confidence intervals (CI) are also displayed

1. NDMM: 2340 patients composed the NDMM intention‐to‐treat (ITT) population from the CASSIOPEIA, MAIA and ALCYONE studies; 1982 patients had standard‐risk disease (SRD) and 358 had HRD. The addition of Dara to backbone therapy reduced the risk of progression or death by 57% (HR 0.43; 95% CI, 0.35‐0.53; Figure 1A) in SRD and by 23% in HRD, however the latter was not statistically significant (HR 0.77; 95% CI, 0.53‐1.11; Figure 1B).
2. RRMM: 673 patients composed the RRMM ITT population from the CASTOR and POLLUX studies; 513 patients had SRD and 160 had HRD. The addition of Dara reduced the risk of progression or death by 72% (HR 0.28; 95% CI, 0.21‐0.36; Figure 1C) in SRD and by 52% (HR 0.48; 95% CI, 0.30‐0.76; Figure 1D) in HRD.

## 4 DISCUSSION

To our knowledge, this is the first meta‐analysis examining the effect of Dara in HRD and SRD MM. In SRD, the addition of Dara provides a clear PFS benefit. In NDMM HRD, the addition of Dara indicates a favorable but not statistically significant PFS benefit. Currently, standard therapy for NDMM is bortezomib (V) with lenalidomide and dexamethasone (RD) that demonstrated superior PFS and OS compared to RD alone [11]. While the small sample size of HRD patients possibly contributed to the lack of statistical significance of the HR, until further data emerge from studies evaluating the addition of Dara to VRD or KRD (carfilzomib with lenalidomide and dexamethasone), we believe VRD remains the important backbone in NDMM HRD.

In RRMM, the addition of Dara led to a clear PFS benefit in patients with SRD with a HR of 0.28. In HRD, the improvement was less striking but still resulted in a significant HR of 0.48. This is comparable to the PFS benefit seen with use of KRD in HRD compared to RD (HR 0.7, *P* < .05) in the ASPIRE trial [12] as well as the ELOQUENT‐2 trial comparing elotuzumab with RD to RD alone with a HR of 0.76 in del(17p) (both *P* < .05) and 0.56 in t(4:14) patients [13]. Overall, the PFS advantage with Dara combinations in the RRMM seems favorable or at least comparable to other regimens commonly used for relapsed disease.

While this study could not definitively show benefit of Dara in ND HRD, the admittedly smaller sample size resulted in wide confidence intervals. Another potential reason is that the relapsed/refractory studies (POLLUX and CASTOR) both have doublets in the control arm, which are inferior to triplets. In NDMM, the CASSIOPEIA and ALCYONE trial control arms are triplets leading to comparable outcomes to a four‐drug combination in HRD. Since patients with NDMM are more chemo‐sensitive regardless of cytogenetic risk, doublets (such a RD) or regimens without either IMIDs or PIs (such as VMP) perform better as compared to the relapsed/refractory patients with more pronounced effects.

In conclusion, our analysis shows that while clearly efficacious in SRD and RR HRD, the role of Dara in ND HRD is less clear. Preliminary data from the GRIFFIN and MASTER trials show higher rates of deep responses and MRD negativity with Dara + VRD compared to VRD alone and high rates of deep responses and MRD negative with Dara + KRD, respectively [14, 15]. Stratification by cytogenetic group is eagerly awaited and will enhance the power of this study, perhaps leading to a definitive answer.

## AUTHOR CONTRIBUTIONS

Dr. Vikram Premkumar and Dr. Divaya Bhutani had full access to all the data in the study and takes responsibility for the integrity of the data and the accuracy of the data analysis. All authors were associated with the study concept and design, acquisition, analysis, and interpretation of data. Premkumar and Bhutani drafted the manuscript. Premkumar, Bhutani, and Lentzsch critically revised the manuscript for important intellectual content. Pan performed the statistical analysis. Bhutani and Lentzsch supervised the study.

## CONFLICTS OF INTEREST

The Conquer Cancer Foundation and the National Institutes of Health had no role in the design and conduct of the study; collection, management, analysis, and interpretation of the data; preparation, review, or approval of the manuscript; and decision to submit the manuscript for publication. VP has no conflicts of interest. SP has no conflicts of interest. SL is a shareholder of Caelum Biosciences and has received consultant fees from Caelum Biosciences, Bayer, AbbVie, Janssen, Proclara, and Takeda and receives research funding from Karyopharm and Sanofi. DB serves on the advisory board for Sanofi.
